# Whole genome sequence and annotation dataset of rare actinobacteria, *Barrientosiimonas humi gen. nov., sp. nov.* 39^T^ from Antarctica

**DOI:** 10.1016/j.dib.2023.109657

**Published:** 2023-10-12

**Authors:** Sin Yee Chong, Aida Azrina Azmi, Yoke Kqueen Cheah

**Affiliations:** a Unit of Molecular Biology and Bioinformatics, Department of Biomedical Science, Faculty of Medicine and Health Sciences, Universiti Putra Malaysia, 43400 UPM Serdang, Selangor Darul Ehsan, Malaysia; b Halal Science Research, Halal Products Research Institute, Universiti Putra Malaysia, 43400 UPM Serdang, Selangor Darul Ehsan, Malaysia; c UPM-MAKNA Cancer Research Laboratory, Institute of Bioscience, Universiti Putra Malaysia, Serdang, Selangor, Malaysia

**Keywords:** Antibacterial, Antifungal, Antarctica, *Barrientosiimonas humi*, Biosynthetic gene clusters, Cytotoxic, Genome, Inhibitor

## Abstract

*Barrientosiimonas humi gen. nov., sp. nov.* 39^T^ is a rare actinobacteria strain isolated from the less explored extreme environment of the Antarctic soil. Here, we present the whole genome sequencing and annotation data from the high-quality draft genome of *B. humi* from Antarctica. The extracted genomic deoxyribonucleic acid (DNA) was sequenced using the PacBio Sequel sequencing platform, followed by the Illumina HiSeq sequencing system. Subsequently, the assembly data from Canu 1.7 and Pilon were subjected to bioinformatics analysis for genome annotation to analyze the entire genomic information of the sequences. Different bioinformatics analysis approaches were used to disclose a high-quality draft genome basis for *B. humi* and provided a better understanding of its biological and molecular functions. Note that 83,639 reads were predicted from its 3.6Mb genome size, with a guanine-cytosine content (GC) content of 72.39%. The genome was assembled into two contigs, where the larger contig represents the chromosome and the smaller contig represents the plasmid. It is composed of 3,381 coding genes, with about 95% of them being functionally annotated. It consists of 3,318 coding sequences, one tmRNA gene, 57 tRNA genes, and five repeated regions. *B. humi* was evident, sharing a close sequence similarity with the species *Demetria terragena* and the family *Dermacoccaceae*. Gene Ontology (GO) functional classification indicated cell and cell parts were highly represented among the cellular component category; catalytic activity and binding were the most enriched processes within the molecular function category; metabolic and cellular processes were the most represented in the biological process category. Clusters of Orthologous Group (COG) functional classification revealed metabolism-related genes were highly enriched and mostly mapped to amino acid transport metabolism, transcription, energy production, and conversion. Moreover, the Kyoto Encyclopedia of Genes and Genomes (KEGG) functional classification reported that the metabolism process was the most represented KEGG pathway. There were 52 biosynthetic gene clusters involved in secondary metabolites biosynthesis, indicating *B. humi* has antibacterial, antifungal, cytotoxic, and inhibitor bioactivities. The dataset of the whole-genome sequence of *B. humi* has been deposited in the European Nucleotide Archive (ENA) repository under the accession number PRJEB44986 / ERP129097. The dataset of the genome annotation of *B. humi* had been deposited in Zenodo. The reported genomic sequence data for *B. humi* contributes comprehensive data to the current molecular information of the species, serving as a significant approach that facilitates the advancement of medicine.

Specifications Table

**Table:** 

Subject	Biology
Specific subject area	Microbiology and Genomics
Type of data	Table Figure
How the data were acquired	**Sequencing platform:** PacBio Sequel sequencing platform and Illumina HiSeq sequencing system **Assembly program:** Canu v1.7 and Pilon **Genome coverage:** 141.25x **Annotation approach:** **Genome assembly and structural annotation:** Quast v3.1, Prokka and BUSCO v2.0 **Functional annotation and classification:** DIAMOND v0.9.22, BLASTX v2.7.1+, and eggNOG-mapper v2.0.0 analysis based on eggNOG v5.0 **Biosynthetic Gene Clusters (BGCs) detection:** antiSMASH 5.0.0, BAGEL 4, and DeepBGC v0.1.14
Data format	Raw Analyzed Assembled Annotated
Description of data collection	*Barrientosiimonas humi gen. nov., sp. nov.* 39^T^ is selected for this research. It is a rare actinobacterial, isolated from the less explored extreme environment of the upper topsoil layer of Antarctic soil (62ͦ 24’ 26” S 59ͦ 44’ 49.1” W) on Barrientos Island during the XI Ecuadorian Antarctic Expedition in 2007. The extracted genomic DNA of *B. humi* was sequenced using the PacBio Sequel sequencing platform and processed for assembly as input to Canu v1.7. The Illumina HiSeq sequencing system was applied. The Illumina paired-end reads and the PacBio consensus reference was processed for assembly. Pilon was used to further correct the sequencing data. The assembly data were then subjected to the genome and functional annotations through Quast v3.1, Prokka, BUSCO v2.0, DIAMOND v0.9.22, BLASTX v2.7.1+ and eggNOG-mapper v2.0.0 based on eggNOG5.0. The secondary metabolites BGCs of *B. humi* genome sequences were predicted by the computational genome mining software tools, which were antiSMASH 5.0.0, BAGEL 4, and DeepBGC v0.1.14
Data source location	• Institution: Instituto Antártico Ecuatoriano (INAE) • City/Town/Region: Barrientos Island • Country: Antarctica • Latitude and longitude (and GPS coordinates, if possible) for collected samples/data: Upper topsoil layer of Antarctic soil (62ͦ 24’ 26” S 59ͦ 44’ 49.1” W)
Data accessibility	Repository name: European Nucleotide Archive (ENA) Data identification number: PRJEB44986 Secondary study accession: ERP129097 Direct URL to data (ENA): https://www.ebi.ac.uk/ena/browser/view/PRJEB44986 Zenodo DOI Number for the annotated genome (source files): 10.5281/zenodo.8265496 Direct URL to data (Zenodo): https://zenodo.org/record/8265496
Related research article	Lee, L., Cheah, Y., Sidik, S. M., Xie, Q., Tang, Y., Lin, H., & Mutalib, N. A. (2013). Actinobacterium of the Family *Dermacoccaceae. International Journal of Systematic and Evolutionary Microbiology, 63*(1), 241–248. https://doi.org/10.1099/ijs.0.038232-0.

## 1 Value of the Data

• Currently, limited studies are focused on isolating antibiotics from rare actinobacterial strains such as *B. humi*. Based on the number of predicted BGCs detected in this strain's high-quality draft genome sequence, the raw dataset can serve as a significant resource for discovering potentially novel bioactive compounds.
• The annotated genome sequences of *B. humi* can provide insights into the genetic basis for the strain's biological and molecular functions.
• The complete whole genome sequences and annotation dataset of rare actinobacteria, *B. humi* represent a great and valuable dataset for the taxonomists (understanding the relatedness of this strain to other members of the *Dermacoccaceae*), as well as the researchers in medical sciences (genome mining for biosynthetic gene clusters), genomics (gene content and distribution), microbiology (the genomic basis for phenotypic properties), and biotechnology fields (genome mining for secondary metabolites, including enzymes).

## 2 Objective

The presented data article is co-related to a research article, “*Barrientosiimonas humi gen. nov., sp. nov.,* an actinobacterium of the family *Dermacoccaceae*”, which was published in the International Journal of Systematic and Evolutionary Microbiology. The presented dataset includes the sequenced and assembled genomic sequences, which have been quality-assessed, and the functionally annotated genes from *B. humi*. On the other hand, whole-genome sequencing technology combined with the bioinformatics approach manages and analyzes the genetic makeup of *B. humi*. The annotated whole-genome sequences of *B. humi* provides a better understanding of its biological and molecular functions. The extreme environment, especially the polar glacier region, notably Antarctica, offers significant potential as a source for rare actinobacteria strains and a promising source for intriguing secondary metabolites. Detecting predicted BGCs in *B. humi* aids in discovering novel bioactive compounds. Hence, the presented dataset is important to serve as a high-quality draft genome basis for future studies.

### 2.1 Data Description

Here, we present the whole genome sequence of *B. humi* obtained from PacBio Sequel and Illumina HiSeq sequencing systems. *B. humi* is selected as the rare actinobacterial strain in this research. *B. humi* was isolated from the less explored extreme environment of the upper topsoil layer of Antarctic soil (62ͦ 24’ 26” S 59ͦ 44’ 49.1” W) on Barrientos Island during the XI Ecuadorian Antarctic Expedition in 2007 [1].

*B. humi s*train 39^T^ forms a monophyletic branch, closely related to *Demetria terragena* HKI 0089T, with a 99% bootstrap value, and shares a high 16S ribosomal ribonucleic acid (rRNA) gene sequence similarity of 96.90%. Apart from these similarities, based on the differences in phylogenetic, chemotaxonomic, phenotypic, and 16S rRNA gene signature nucleotide analyses, strain 39^T^ is remarkably distinct from all other genera in the *Dermacoccaceae* family. Therefore, strain 39^T^ is designated to the novel genus in the family *Dermacoccaceae, Barrientosiimonas gen. nov.*, specifically the type species known as *Barrientosiimonas humi gen. nov., sp. nov.* [1]. The genus name *Barrientosiimonas* was legitimately published early in 2013 with *Barrientosiimonas humi* 39^T^ as the type [1]. In 2015, based on the results from phylogenetic, polyphasic taxonomic, and 16S rRNA gene signature nucleotide studies, strain JC268^T^ displayed strong 16S rRNA gene sequence similarity to *Barrientosiimonas humi* 39^T^ (98.65%) and *Tamlicoccus marinus* MSW-24^T^ (97.80%) in the family *Dermacoccaceae*. It is also assigned to the novel species of the genus *Barrientosiimonas,* known as *Barrientosiimonas endolithica sp. nov.* [2]. Meanwhile, the data proposes the reclassification of *T. marinus* within the genus *Barrientosiimonas* as *Barrientosiimonas marina comb. nov.*, which entailed the emendation of the genus *Barrientosiimonas* description [2].

The genome features of *B. humi* are summarized in Table 1. Through the genome assembly by Canu 1.7, Pilon and Quast v3.1, 83,639 reads for *B. humi* were assembled into two contigs (more than 1000bp). Here, the larger contig represented the chromosome (3.6Mb) while the smaller contig (7kb) represented the plasmid, with a total genome size of approximately 3.6Mb. The largest contig length of *B. humi* is 3,615,725kb, with a total length of 3,622,728 bases in the assembly. Through the genome assembly completeness assessment using BUSCO v2.0, the assembled genomes were discovered to have a high degree of completeness of 99.4% (Fig. S1). Through the subsequent structural annotation by Prokka and BUSCO v2.0, a 3.6Mb genome of *B. humi*, assembled from 3,381 coding genes, was formed. *B. humi* genome consists of 3,318 coding sequences (CDS), one transfer-messenger ribonucleic acid (tmRNA) gene, 57 transfer ribonucleic acid (tRNA) genes, and five repeated regions without the presence of rRNA operons. A high percentage of completeness of 99.2% was reached for both the transcriptome and gene set according to the BUSCO v2.0 assessments (Fig. S1).

**Table 1:** Genome features of *B. humi*.

Genomic features	Value
**Assembly assessment**	
# contigs (≥300 bp)	2
# contigs (≥500 bp)	2
# contigs (≥1000 bp)	2
Total length (≥300 bp)	3622728
Total length (≥500 bp)	3622728
Total length (≥1000 bp)	3622728
Contigs no.	2
Largest contig	3615725
Total length	3622728
GC content (%)	72.39
N50	3615725
L50	1
L75	1
# N's per 100 kbp	0
BUSCO completeness for genome assembly profile	C:99.4%[S:99.4%,D:0.0%],F:0.3%,M:0.3%,n:352
	
**Structural annotation assessment**	
Total number of genes	3381
Protein coding sequences (CDS)	3318
rRNA	0
tmRNA	1
tRNA	57
Repeat region	5
BUSCO completeness for transcriptome profile	C:99.2%[S:98.3%,D:0.9%],F:0.3%,M:0.5%,n:352
BUSCO completeness for gene set profile	C:99.2%[S:98.3%,D:0.9%],F:0.3%,M:0.5%,n:352

*Note:* N50 described the contig length, whereas L50 and L75 corresponded to the number of contig, which covered at least 50% and 75% of the genome assembly size, respectively. N's represented the uncalled bases, which were the unqualified bases.

The genome sequences derived from *B. humi* were subjected to functional annotation. The top hit had high coverage of 95.42% and 71.40% with respect to the NCBI RefSeq protein database and SP protein databases via DIAMOND v0.9.22 and BLASTX v2.7.1+ (BLASTX search via NCBI's BLAST+) (Fig. S2). *B. humi* peptide sequences were also subjected to the search in the eggNOG v5.0 orthology database. The result showed 93.37% high coverage of proteins in the database (Fig. S3). The best-aligning results were selected for further annotation. Approximately 95% of annotated coding genes were functionally annotated and had relatively well-conserved functions.

A top-hit species distribution analysis based on the BLASTX result was carried out. Among the RefSeq top-hit species, *B. humi* demonstrated the strongest similarity to the sequences of *Demetria terragena* (1,186/3,166, 37.46%) (Fig. S4). *B. humi* had the highest degree of sequence similarity to the sequences of *Demetria terragena*, following the finding by Lee et al. [1]. From the eggNOG 5.0 orthology database, taxonomic group distribution analysis based on the eggNOG v5.0 seed ortholog revealed that *B. humi* had the highest degree of sequence similarity with the sequences of the family *Dermacoccaceae* (1,591/3,098, 51.36%) (Fig. S5). The sequences of *B. humi* had the highest similarities to the *Dermacoccaceae* family, in line with the previous study [1].

**Fig. 1:**
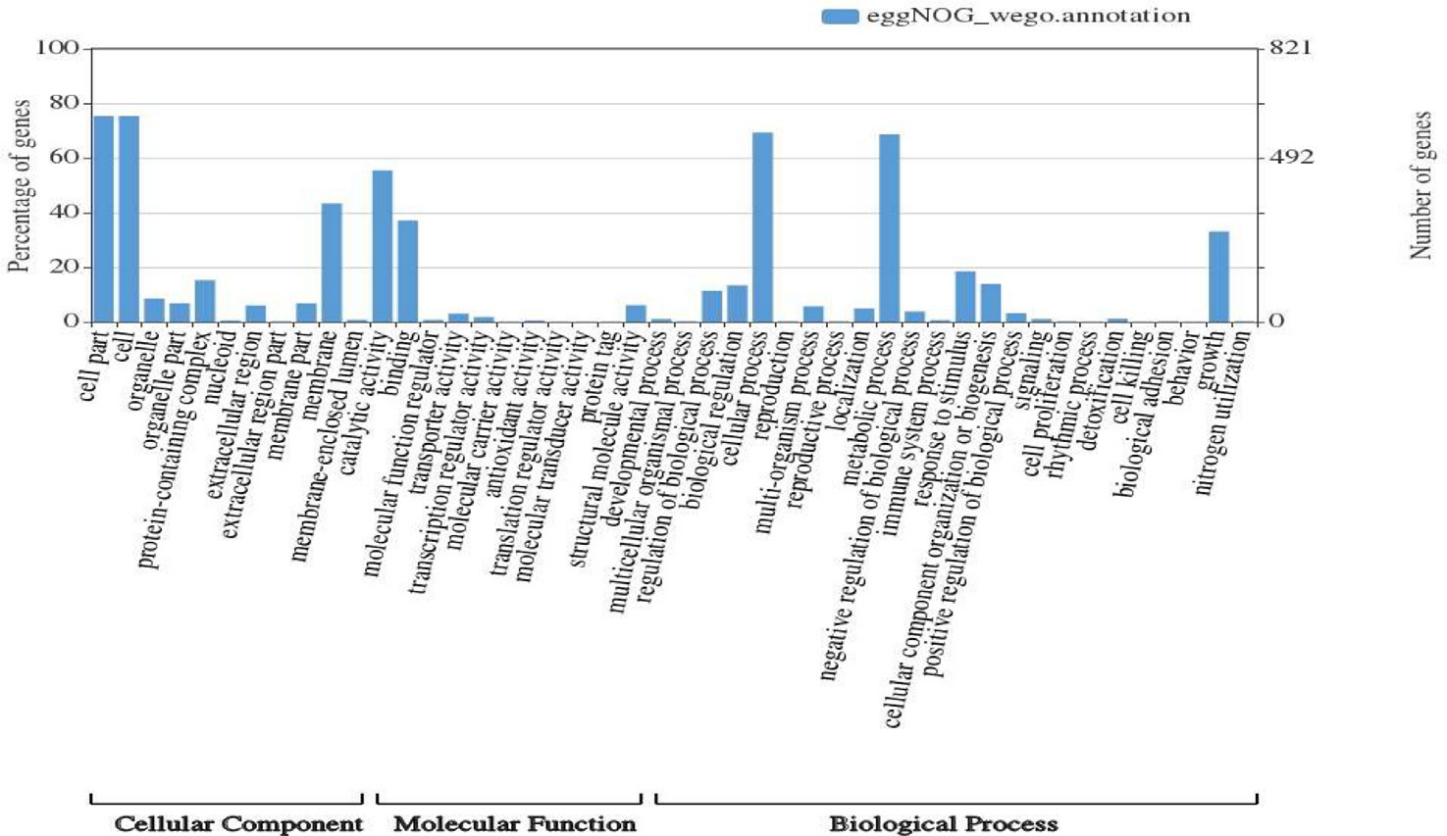
GO functional classification analysis using eggNOG-mapper. GO terms were assigned at GO annotation level 2. The results were summarized in terms of three functional groups, namely, cellular component, molecular function, and biological process. The right y-axis indicates the number of genes in a category. The left y-axis indicates the percentage of a specific category of genes in that main category.

The GO functional classification of *B. humi* shows that there are 46 functional groups within the three main categories, which are assigned as cellular component (11), molecular function (11), and biological process (24). For the cellular component category, GO terms most noticeably correspond to cell (618 genes, 75.3%) and cell part (618 genes, 75.3%). In tems of molecular function, most genes act on catalytic activity (456 genes, 55.5%) and binding (305 genes, 37.1%). For the biological process category, GO terms correspond highly to the cellular process (569 genes, 69.3%) and metabolic process (564 genes, 68.7%) (Fig. 1; Table S1).

**Fig. 2:**
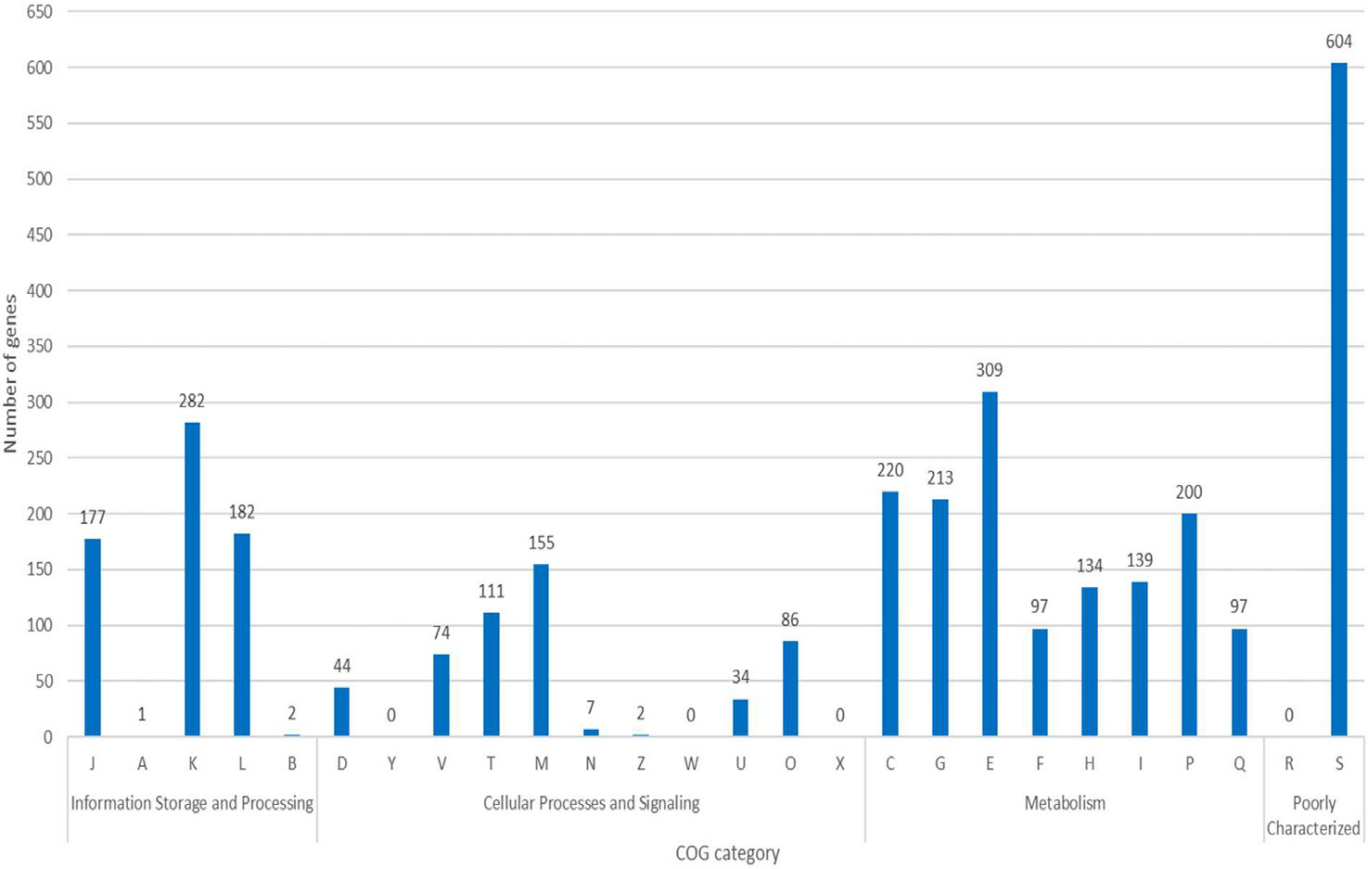
COG functional classification analysis among 25 categories using eggNOG-mapper.

To further evaluate the gene function of *B. humi* against various functional classes from the COG database, the majority of *B. humi* genes were functionally divided into three categories: information storage and processing, cellular processes and signaling, and metabolism. The genes with unclassified functions were categorized into poorly characterized groups. The genes were then clustered into twenty-five COG functional classes*.* Overall, the majority of genes (2,566 genes, 80.95%) were conferred with attributable functions. Of these, most genes (1,409 genes, 44.45%) were mainly enriched for metabolism processes, followed by about a quarter of genes (644 genes, 20.32%) were found to be involved in information storage and signaling. Comparatively, the fewest genes (513 genes, 16.18%) were associated with cellular processes and signaling*.* From this COG functional classification analysis, most of the genes in *B. humi* were mapped to amino acid transport metabolism (309 genes, 9.75%), followed by transcription (282 genes, 8.90%) and energy production and conversion (220 genes, 6.94%) (Fig. 2; Table S2).

**Fig. 3:**
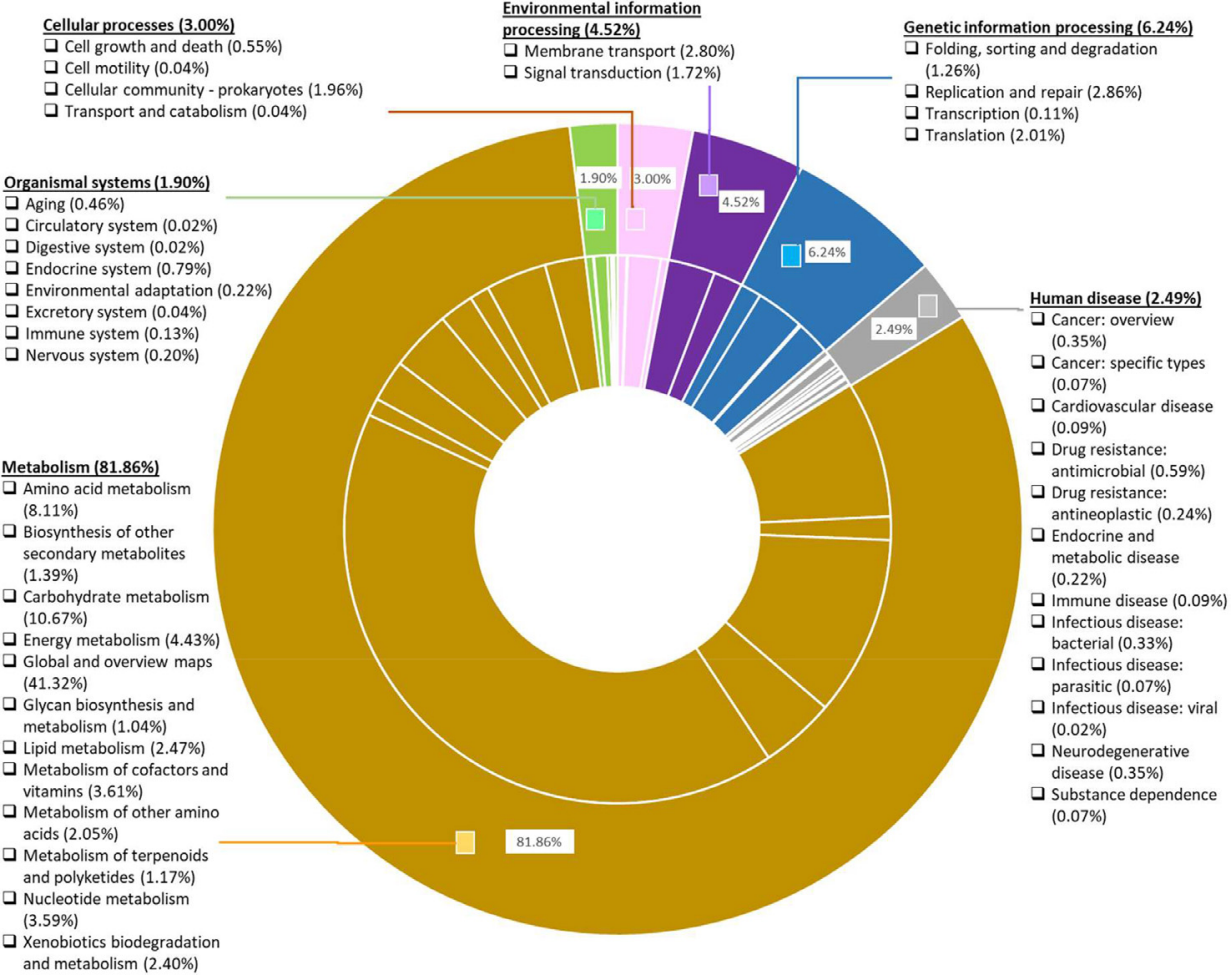
KEGG functional classification of *B. humi* by the number of mapped enzymes using eggNOG-mapper. The KEGG pathways were divided into six classifications, which were cellular processes, environmental information processing, genetic information processing, human diseases, metabolism, and organismal systems.

Consequently, the KEGG functional classification depicts that 1,231 *B. humi* assembled sequences are mapped to six main functional categories against the KEGG database for 243 KEGG pathways (Fig. 3; Table S3). The most represented KEGG pathways category is metabolism processes (3,715/4,538, 81.86%), which is divided into 12 subcategories. This is followed by genetic information processing (283/4,538, 6.24%), which consists of four subcategories and environmental information processing (205/4,538, 4.52%), which has two subcategories*.* Among the metabolism processes category, the maximum concentration of genes in metabolism is in global and overview metabolism (1,875/3,715, 41.32%), followed by carbohydrate metabolism (484/3,715, 10.67%) and amino acid metabolism (368/3,715, 8.11%). J, Translation, ribosomal structure and biogenesis; A, RNA processing and modification; K, Transcription; L, Replication, recombination and repair; B, Chromatin structure and dynamics; D, Cell cycle control, cell division, chromosome partitioning; Y, Nuclear structure; V, Defence mechanisms; T, Signal transduction mechanisms; M, Cell wall/membrane/envelope biogenesis; N, Cell motility; Z, Cytoskeleton; W, Extracellular structures; U, Intracellular trafficking, secretion, and vesicular transport; O, Posttranslational modification, protein turnover, chaperones; X, Mobilome: prophages, transposons; C, Energy production and conversion; G, Carbohydrate transport and metabolism; E, Amino acid transport and metabolism; F, Nucleotide transport and metabolism; H, Coenzyme transport and metabolism; I, Lipid transport and metabolism; P, Inorganic ion transport and metabolism; Q, Secondary metabolites biosynthesis, transport and catabolism; R, General function prediction only; S, Function unknown.

The secondary metabolites BGCs of *B. humi* genome sequences were predicted using antiSMASH 5.0.0. A total of 17 secondary metabolites BGCs regions were retrieved from tig00000001_arrow_pilon (Table 2A). BAGEL 4 enables the observation of tig00000001_arrow_pilon, a single area of interest (AOI) found from *B. humi* sequences (Table 2B). DeepBGC v0.1.14 predicts a total of 34 gene clusters in tig00000001|arrow|pilon. *B. humi* is observed to have antibacterial, antifungal, cytotoxic, and inhibitor activities (Table 2C). By utilizing three computational genome mining approaches, as shown in Table 2, a total of 52 BGCs regions engaged in secondary metabolites or natural product biosynthesis were predicted in tig00000001_arrow_pilon (chromosome), however none were detected in tig00000030_arrow_pilon (plasmid). *B. humi* is identified to have antibacterial, antifungal, cytotoxic, and inhibitor metabolites BGCs regions.

**Table 2:** Secondary metabolites BGCs regions in *B. humi*. (A) Identified BGCs regions by antiSMASH 5.0.0. (B) Identified AOI by BAGEL 4. (C) Predicted BGCs product class and potential chemical activity by DeepBGC v0.1.14.

(A) Identified BGCs regions by antiSMASH 5.0.0.					
Region	Type	Level*	Fasta header	From	To
Region 2.1	ectoine	I	tig00000001_arrow_pilon	218,208	228,597
Region 2.2	saccharide	III	tig00000001_arrow_pilon	274,395	307,828
Region 2.3	halogenated	III	tig00000001_arrow_pilon	681,455	702,762
Region 2.4	saccharide	III	tig00000001_arrow_pilon	1,160,125	1,193,871
Region 2.5	saccharide	III	tig00000001_arrow_pilon	1,251,942	1,282,901
Region 2.6	LAP	I	tig00000001_arrow_pilon	1,300,865	1,322,848
Region 2.7	saccharide	III	tig00000001_arrow_pilon	1,464,860	1,488,149
Region 2.8	saccharide	III	tig00000001_arrow_pilon	1,921,529	1,956,776
Region 2.9	saccharide	III	tig00000001_arrow_pilon	2,006,178	2,049,547
Region 2.10	saccharide	III	tig00000001_arrow_pilon	2,222,013	2,248,504
Region 2.11	fatty_acid	III	tig00000001_arrow_pilon	2,258,925	2,280,001
Region 2.12	siderophore	I	tig00000001_arrow_pilon	2,428,914	2,441,250
Region 2.13	saccharide	III	tig00000001_arrow_pilon	2,699,808	2,764,547
Region 2.14	saccharide	III	tig00000001_arrow_pilon	2,837,541	2,909,471
Region 2.15	saccharide	III	tig00000001_arrow_pilon	2,914,934	2,937,463
Region 2.16	saccharide	III	tig00000001_arrow_pilon	2,938,924	2,960,098
Region 2.17	saccharide, NRPS-like	II	tig00000001_arrow_pilon	3,258,812	3,302,933

Note: Detection strictness level: I – strict, i.e. well-defined clusters containing all required parts. II – relaxed, i.e. partial clusters missing one or more functional parts. III – i.e. poorly-defined clusters and clusters that likely match primary metabolites. There were 17 secondary metabolites BGCs regions observed in tig00000001|arrow|pilon (chromosome), without any BGC found in tig00000030_arrow_pilon (plasmid).					
**(B)** Identified AOI by BAGEL 4.					
AOI	Type	Fasta header	From	To	

AOI_01	Sactipeptides	tig00000001_arrow_pilon	384,953	404,953	

Note: There was one AOI in tig00000001|arrow|pilon (chromosome), without any AOI found in tig00000030_arrow_pilon (plasmid).					
**(C)** Predicted BGCs product class and potential chemical activity by DeepBGC v0.1.14.					
No	Chemical activity	Compound class	Fasta header	From	To

1			tig00000001|arrow|pilon	33,769	46,163
2			tig00000001|arrow|pilon	55,376	62,194
3	antibacterial		tig00000001|arrow|pilon	207,641	209,054
4	antibacterial	Other	tig00000001|arrow|pilon	221,862	224,603
5	antibacterial		tig00000001|arrow|pilon	590,660	591,275
6	cytotoxic	Polyketide	tig00000001|arrow|pilon	689,236	692,762
7	antibacterial	Saccharide	tig00000001|arrow|pilon	1,260,605	1,262,976
8	antibacterial	Saccharide	tig00000001|arrow|pilon	1,264,371	1,270,855
9	antibacterial		tig00000001|arrow|pilon	1,809,560	1,818,634
10	antibacterial		tig00000001|arrow|pilon	1,819,615	1,820,194
11	antibacterial	Polyketide	tig00000001|arrow|pilon	2,016,018	2,016,768
12		Saccharide	tig00000001|arrow|pilon	2,018,827	2,049,547
13		Saccharide	tig00000001|arrow|pilon	2,222,012	2,246,063
14		Polyketide	tig00000001|arrow|pilon	2,259,791	2,274,654
15	antibacterial	NRP	tig00000001|arrow|pilon	2,281,476	2,282,289
16			tig00000001|arrow|pilon	2,285,195	2,291,092
17			tig00000001|arrow|pilon	2,374,414	2,383,140
18	antibacterial		tig00000001|arrow|pilon	2,397,385	2,397,874
19	antibacterial		tig00000001|arrow|pilon	2,431,859	2,432,630
20	antibacterial		tig00000001|arrow|pilon	2,547,776	2,549,086
21		Other	tig00000001|arrow|pilon	2,568,308	2,580,433
22	antibacterial-cytotoxic		tig00000001|arrow|pilon	2,713,219	2,754,841
23	antibacterial	Saccharide	tig00000001|arrow|pilon	2,855,608	2,917,481
24	antibacterial		tig00000001|arrow|pilon	2,927,278	2,927,890
25	antibacterial	Polyketide	tig00000001|arrow|pilon	3,095,628	3,097,514
26	antibacterial-antifungal	Polyketide	tig00000001|arrow|pilon	3,443,589	3,444,804
27	antibacterial	Polyketide	tig00000001|arrow|pilon	3,445,483	3,446,770
28			tig00000001|arrow|pilon	3,447,935	3,470,905
29			tig00000001|arrow|pilon	3,498,572	3,499,319
30	cytotoxic		tig00000001|arrow|pilon	3,521,185	3,530,797
31	antibacterial		tig00000001|arrow|pilon	3,575,597	3,582,367
32			tig00000001|arrow|pilon	3,583,987	3,587,490
33	inhibitor		tig00000001|arrow|pilon	3,593,448	3,595,002
34			tig00000001|arrow|pilon	3,599,504	3,615,725

Note: There were 34 BGCs observed in tig00000001|arrow|pilon (chromosome), without any BGC found in tig00000030_arrow_pilon (plasmid).

## 3 Experimental Design, Materials and Methods

### 3.1 *B. humi* sample and genome DNA extraction

*B. humi* is a rare actinobacteria maintained as a laboratory strain. It was isolated from the less explored extreme environment of the upper topsoil layer of Antarctic soil (62ͦ 24’ 26” S 59ͦ 44’ 49.1” W) on Barrientos Island during the XI Ecuadorian Antarctic Expedition in 2007. The procedures for *B. humi* isolation, incubation, and DNA extraction were previously described in the study by by Lee et al. [1].

### 3.2 Genome sequencing

The genomic DNA was sheared to ∼15-20 kb using the Megarupter (Diagenode). The PacBio Microbial Multiplexing Calculator was used to calculate the DNA amount needed for each sample based on genome size and shearing. The entire volume was used for pooling after the ligation step. The PacBio library DNA underwent size selection with a 7 kb cut-off, using a Pippin Prep (Sage Science). A final 0.45 X Ampure clean-up was performed, resulting in an 11 ul volume. The extracted genomic DNA was then sequenced using the PacBio Sequel sequencing platform, with the size selection for the fragments longer than 9kb [3]. Subsequently, the qualified DNA was cut into fragments by the restriction enzyme. The Illumina DNA libraries were constructed through end repairing, adding A to tails, purification, and PCR amplification. Note that Illumina libraries were sequenced using a high-throughput sequencer with a paired-end sequencing strategy on the Illumina HiSeq sequencing system, with an insert size of 500bp [4].

### 3.3 Genome assembly

The PacBio sequence reads were initially converted from the trimmed BAM format file to FASTA using BBMap reformat. Subsequently, these sequences were processed for assembly as input to Canu v1.7, with parameter ‘corOutCoverage’ set to 60. The assembly data from Canu 1.7 produced a read depth of 141.25 over the genome [5]. The Illumina paired-end 150 bp reads were trimmed using BBDuk then aligned with BBMap, resulting in a BAM file. The resulting BAM file, along with the PacBio consensus reference were processed for assembly, and then used as input to Pilon for further error correction of the reads [6].

### 3.4 Genome annotation

The assembly data were analysed using bioinformatics tools to extract the entire genomic information from the sequences. The assembly data underwent structural annotation using Quast v3.1 [7] and Prokka [8]. The genome assembly, transcriptome, and gene set completeness assessment were then assessed using BUSCO v2.0 [9,10]. For functional annotation, *B humi* annotated peptide sequences were subjected to a search against the NCBI Reference System (RefSeq) protein database, Swiss-Prot (SP) protein database using DIAMOND v0.9.22 and BLASTX v2.7.1+ (BLASTX search via NCBI's BLAST+) [11,12]. Additionally, the sequences were compared to the eggNOG v5.0 orthology database to analyze the protein coverage. Top-hit species distribution analysis was performed based on BLASTX results. Meanwhile, the taxonomic group distribution analysis was performed based on eggNOG v5.0 seed ortholog. Annotated peptide sequences were subsequently subjected to GO, COG, and KEGG functional classification analysis of using eggNOG-mapper v2.0.0 based on eggNOG v5.0 [13,14]. The visualization of GO distribution was performed using WEGO 2.0 [15]. The BGCs of *B. humi* genome sequences were predicted by the computational genome mining software tools, which were antiSMASH 5.0.0 [16,^17^,18], BAGEL 4 [18,19], and DeepBGC v0.1.14 [18].

## Ethics Statements

This work did not involve any animals or human subjects. The authors declare that the manuscript represents the authors’ original work, which has not been published elsewhere. The presented data is complying with the journal's policy.

## CRediT authorship contribution statement

**Sin Yee Chong:** Investigation, Conceptualization, Methodology, Data curation, Writing – original draft, Writing – review & editing. **Aida Azrina Azmi:** Conceptualization, Methodology. **Yoke Kqueen Cheah:** Investigation, Conceptualization, Methodology, Supervision, Writing – review & editing.

## Data Availability

Complete Genome Sequence of Barrientosiimonas humi gen. nov., sp. nov. 39T (Original data) (European Nucleotide Archive)

## Appendix Supplementary materials

Image, application 1
